# Oyster Mushroom Cultivation on Coffee Parchment and *Cenchrus fungigraminus*: A Comparison of Disinfection Methods

**DOI:** 10.3390/jof12060432

**Published:** 2026-06-12

**Authors:** Ben Menda Ukii, Fuke Hako, Abdelnasser Taher, Weizhen Huang, Lin Hui, Yulong Zhang, Zhanxi Lin, Dongmei Lin

**Affiliations:** 1 College of Life Science, Fujian Agriculture and Forestry University, Fuzhou 350002, China; ukiibenmenda@gmail.com (B.M.U.); 12331014079@fafu.edu.cn (F.H.); abdelnassert115@gmail.com (A.T.); 52305043044@fafu.edu.cn (W.H.); yulong900426@163.com (Y.Z.); lzxjuncao@163.com (Z.L.); ldm@fafu.edu.cn (D.L.); 2 National Engineering Research Center of Juncao Technology, International College of Juncao Science, Fujian Agriculture and Forestry University, Fuzhou 350002, China

**Keywords:** *Pleurotus ostreatus*, disinfection methods, coffee parchment, Juncao grass, smallholder farmers, biological efficiency, heavy metals, metabolomics, Papua New Guinea

## Abstract

Conventional sterilization methods limit smallholder mushroom cultivation in PNG. This study evaluated alternative disinfection approaches for *Pleurotus ostreatus* (*P. ostreatus*) using coffee parchment and *Cenchrus fungiraminus* (*C. fungigraminus*) as substrates. After screening 20 strains, the superior strain PXF9 was selected. Three methods were compared: (1) Complete Sterilization with Aseptic Inoculation (CSAI) applied to T1 (experimental) and T2 (sawdust control); (2) Short Sterilization with Open Inoculation (SSOI) applied to T3 (experimental) and T5 (control); and (3) Non sterilization with Open Inoculation (NSOI) applied to T4 (experimental). CSAI (T1) achieved the highest yield (3985.26 ± 2.00 ^d^ g/24 bags), biological efficiency (83.03%), protein (28.44 g/100 g), and profit (14.76 USD), with the fastest colonization (21 days). SSOI (T3) produced the largest fruiting bodies; NSOI (T4) had the lowest heavy metal levels. SSOI and NSOI were economically beneficial (9.88 and 5.96 UDS per 24 bags). Bioactive compounds (e.g., naringenin, ergosterol peroxide), were detected across treatments. While CSAI maximizes productivity, SSOI and NSOI offer low-cost alternatives for resource-limited farmers.

## 1. Introduction

Mushrooms, particularly those of the genus *Pleurotus* (oyster mushrooms), hold significant nutritional and economic value globally due to their ability to convert lignocellulosic wastes into protein-rich food [1,2]. Substrate disinfection is a critical step in mushroom cultivation, as it eliminates competing microorganisms that can otherwise reduce yields. Most previous research has focused on sterilization methods because they achieve lower contamination rates and higher biological efficiency [3,4,5]. This method involves bagging the substrate, autoclaving at 121 °C for 90–160 min, followed by aseptic spawn inoculation [4]. Ali et al. [6] further demonstrated that substrate pretreatment via sterilization eliminates competitive microorganisms during the mycelial run stage. Although this method is effective and efficient, it is costly for rural and small-scale farmers due to limited investment, infrastructure, and skilled personnel [7].

In contrast, non-sterilization is less costly and involves mixing substrates, soaking them in lime water, and bagging. However, this method is prone to contamination and therefore requires more spawn during inoculation [8]. Yang et al. [9] reported no significant difference between non-sterilization and sterilization methods during the mycelial run to fruiting stage. However, formula adjustment significantly improved the non-sterilization method in promoting mycelial run and primordia formation, though it resulted in lower yield and biological efficiency compared to sterilization [9]. Sánchez [10] recommended pasteurization or short sterilization due to lower costs, reporting moderate outcomes. Furthermore, Velázquez Cedeño, Mata, and Savoie [11] successfully cultivated *P. ostreatus* and three strains of *P. pulmonarius* using pasteurization at 80 °C. Atila [12], when evaluating different disinfection methods, reported that chemical disinfection resulted in the greatest biological efficiency, followed by pasteurization at 80 °C and sterilization.

Environmental factors, including temperature, humidity, ventilation, and illumination, regulate the growth and development of oyster mushrooms. Mycelial colonization occurs optimally between 25 °C and 30 °C, while the fruiting stage requires cooler temperature (13–21 °C) [13,14]. Oyster mushrooms tolerate slightly acidic to alkaline conditions, with an ideal pH range of 5.5–7.5 [15,16,17]. Optimal substrate moisture content for mycelial colonization is 60–70%, with relative humidity (RH) of 85–90% during the fruiting [13,14]. Ventilation is essential to keep carbon dioxide concentrations above 600 ppm, as higher levels cause malformation of fruiting bodies. Illumination of 200 lux is required for primordia initiation, with 50–500 lux recommended for 12 h daily [13,17].

Oyster mushrooms favor lignocellulosic substrates such as agricultural waste. Coffee husk, including parchment, is a byproduct of coffee bean processing and possesses significant lignocellulosic properties, containing approximately 52.9% cellulose, 12.5% hemicellulose, and 24.3% lignin, making it a potential substrate for oyster mushroom cultivation [13]. However, it is a major waste product leading to environmental pollution in coffee-producing countries [11]. In Papua New Guinea (PNG), coffee parchment is primarily burned or disposed of, causing significant environmental pollution. Hence, upcycling it as an oyster mushroom substrate will reduce the associated environmental issues. Giant Juncao grass, scientifically known as *Cenchrus fungigraminus*, is a C4 grass that grows rapidly, reaching five meters within six months. It can supply approximately 225–450 tons per hectare annually and lasts for six to seven years, with the potential to withstand and adapt to harsh conditions while providing rich nutrient content [14]. Both substrates are abundant in PNG at no cost.

Oyster mushrooms are recognized as functional foods with high-quality protein rich in essential amino acids, comparable to animal meat [18,19,20,21]. They are low in fat and contain beneficial carbohydrates, including β-glucans, which exhibit immunomodulatory, anti-inflammatory, and anticancer properties, while also improving gut health and cholesterol management [22]. Dietary fiber varies between 23.63% and 52.31% of dry weight depending on the substrate, cultivation conditions, species, and environmental factors [23]. Mineral content is similarly substrate-dependent, with typical ash content ranging from 4.9 to 9.8% dry weight [21,24,25].

Mushrooms can accumulate toxic heavy metals from their substrates, including mercury (Hg), chromium (Cr), cadmium (Cd), lead (Pb), and arsenic (As), which pose health risks such as organ damage, cancer, and neurological effects [26,27,28,29,30,31,32]. Permissible levels set by organizations such as the European Union (EU) and the World Health Organization (WHO) for Pb, Cd, Hg, and As in food range from 0.01 to 3 mg/kg, 0.05–2 mg/kg, 0.05–1 mg/kg, and 0.1–0.2 mg/kg, respectively [32]. Therefore, while mushrooms offer numerous health benefits, proper analysis of the fruiting body is essential before consumption.

This study aims to evaluate different disinfection methods for cultivating *P. ostreatus* using coffee parchment and *C. fungigraminus*, and to recommend suitable methods for rural and local farmers.

## 2. Materials and Methods

### 2.1. Substrate Specification and Formulation

Twenty strains of *P ostreatus* were provided by the China National Engineering Research Center of Juncao Technology (CNERCJT) at Fujian Agriculture and Forestry University (FAFU), Fuzhou, China. The strains were as follows: PXt, P969, PDHP, PXF9, PXF2, PYH8, PXHP, PtP2, P615, P246, PTCHP, PX50, P313, PY9NS, P7448, PFP, PJPg, PZQ615, PXF11, and PX25. Mature, fresh *C. fungigraminus* was harvested, ground to 1–5 mm particles using a laboratory grinder (DF-15, Zhejiang Horus Industry Co., Ltd., Jinhua, China), and sun-dried for seven days until completely dry. Dried coffee parchment was purchased online from Yunnan Province. Sawdust, wheat bran, and lime were obtained from the CNERCJT facility at FAFU, Fuzhou (Supplementary Figure S1).

Five substrate formulations were prepared for three cultivation methods and strain screening (Table 1). T1, T3, and T4 used *C. fungigraminus* and coffee parchment as the primary lignocellulosic base, while T2 and T5 used sawdust. CSAI was applied to T1 (experimental) and T2 (control). SSOI was applied to T3 (experimental) and T5 (control), with T5 serving as the sawdust-based control for strain selection under SSOI. NSOI was applied to T4 (experimental). Moisture content was adjusted to 65–70% using the palm/squeeze test [4].

For strain selection, the SSOI method (T3 formulation) served as the screening platform. Test tubes were filled to 8/10 capacity with substrate, inoculated with 3–5 g of secondary spawn from each of the 20 strains (in triplicate), sealed with breathable caps, and incubated in a dark room at 25 °C. Mycelial growth length was measured weekly for six weeks. Based on growth performance (Section 3.1), *P. ostreatus* PXF9 was selected for all subsequent disinfection method experiments.

### 2.2. Preparation of Primary Spawn and Multiplication of Secondary Spawn

Potato Dextrose Agar (PDA) was prepared by washing, weighing (200 g) and slicing potatoes into small pieces. The slices were boiled in 1 L of water for 30 min, then filtered through cheesecloth to obtain potato infusion. Dextrose (20 g) and agar (20 g) were added to the infusion, mixed, and boiled for another 30 min. The solution was poured into two 500 mL conical flasks, each containing approximately 250 mL of the medium. The flasks were wrapped with paper and a rubber band (Supplementary Figure S2 and Table S1) and sterilized at 121 °C for 20 min using an autoclave (LDZX-75KBS, Shanghai Shenan Medical Instrument Factory, Shanghai, China). After sterilization, 20–25 mL portions were dispensed into sterile Petri dishes ([15 × 100 mm], Jiangsu Huida Medical Instruments Co., Ltd., Yancheng, China) and allowed to cool to room temperature. Subsequently, 3–5 g of spawn strain PXF9 were aseptically inoculated onto the PDA as primary spawn [33]. The dishes incubated in darkness at 25 °C, achieving full colonization after 7 days. The primary spawn on PDA was then used to inoculate sterilized Juncao substrate to produce secondary spawn, following Claude et al. [4].

### 2.3. Common Techniques in All Disinfection Methods

All substrates were adjusted to 65–70% moisture content. This was assessed using the palm/squeeze test, whereby a handful of substrate was squeezed for three to four seconds until a few drops appeared between the fingers [4]. For each treatment, 500 g of substrate was packed in polyethylene bags (25 cm × 50 cm) with 30 replicates. After spawn inoculation, perforated caps were sealed onto the bags for aeration. For T3, T4, and T5, 3-spawn was inoculated at 3–5%. All samples were incubated at 25–26 °C with 70–80% relative humidity. Twenty-four bags per treatment were used for data collection, while the remaining 6 bags served as a contingency for contamination or handling losses.

### 2.4. Substrate Disinfection via CSAI Method (T1 and T2)

For T1, substrates were mixed together with water to achieve the target moisture content. For T2, sawdust was soaked in water for 12 h to absorb moisture, then removed and mixed with supplements. Each substrate was packed into polyethylene bags, sealed with perforated caps, and sterilized at 121 °C for 120 min. The bags were then transferred to an aseptic inoculation chamber [4]. After 24 h, 10 g of secondary spawn were inoculated, followed by incubation (Supplementary Figure S3).

### 2.5. Substrate Disinfection via SSOI Method (T3 and T5)

In the SSOI method, substrates were mixed with water, packed into polyethylene bags and sealed with perforated caps, and autoclaved at 121 °C (15 psi) for 15 min. After cooling for 24 h, 2% lime was added and mixed homogeneously. The substrates were then repacked, inoculated, and incubated (Supplementary Figure S4). This method was applied to the experimental formulation (T3) and the sawdust-based control (T5). This method was applied to both the experimental formulation (T3) and the sawdust-based control (T5).

### 2.6. Substrate Disinfection via NSOI Method (T4)

In this method, 2% lime was first mixed with water in a bucket. Dried *C. fungigraminus* and coffee parchment were then added at a 1:5 ratio (substrates to lime water) and briefly immersed to reduce resident microbes and neutralize antimicrobial compounds [8]. After 12 h, excess water was removed using a string net, and a hydraulic jack squeezed out the remaining water. Supplements were added and mixed thoroughly. The mixture was then packed into polyethylene bags, inoculated, and incubated (Supplementary Figure S5).

### 2.7. Growth and Development Parameters

The inoculated samples (T1–T5) were kept in darkness at 25 °C and 70–80% RH until fully colonized. Weekly assessments recorded the time to mycelial growth, colonization, primordial initiation and harvesting. Flush weights, pileus diameter, stipe length, and biological efficiency (BE) were documented following Claude et al. [4]. BE was calculated as (fresh weight of fruiting body/substrate dry weight) × 100, according to Aimable et al. [5] (Supplementary Figure S6).

### 2.8. Determination of Nutrient Content

Nutritional components were determined according to the National Food Safety Standards of China. Protein content was analyzed using the Kjeldahl method (GB/T 5009.10 2003) [34]. Total carbohydrates were measured by the DNS colorimetric method [35], and total polysaccharides by the phenol-sulfuric acid method [36]. Moisture content was determined gravimetrically (HJ 613 2011) [37], and fat content by the Soxhlet extraction method GB 5009.6 2016 [38]. Crude fiber content was determined using the gravimetric method (GB/T 5009.10 2003) [39], and total ash according to GB 5009.4 2016 [40].

### 2.9. Determination of Heavy Metals

Heavy metals concentrations were determined using inductively coupled plasma mass spectrometry (ICP-MS, iCAP RQ, Thermo Fisher Scientific Inc., Waltham, MA, USA) according to Chinese national standard GB 5009.268 2016 [41]. Elements were identified by their specific mass-to-charge ratios (*m*/*z*) and quantified via an external calibration with an internal standard to ensure accuracy.

### 2.10. Determination of Enzyme Activities

Laccase, manganese peroxidase (MnP), cellulase, xylanase, and alpha-amylase activities were measured at four distinct growth stages: post-inoculation (10 days for T1 and T2; 14 days for T3 and T4), mycelium maturation (21 days for T1 and T2; 28 days for T3 and T4), primordia formation (30 days for T1 and T2; 38 days for T3; 41 days for T4), and fruiting body stage (34 days for T1 and T2; 42 days for T3; 45 days for T4). All assays used commercial kits (Beijing Solarbio Science & Technology Co., Ltd., Beijing China.) following standard spectrophotometric protocols a UV-Vis spectrophotometer (UV-1800, Shimadzu Corporation, Kyoto, Japan).

### 2.11. Metabolite Extraction and LC-MS/MS Analysis

Approximately 25 mg of lyophilized mushroom fruiting body was placed in a 2 mL centrifuge tube. Metabolites were extracted using 1000 μL of pre-cooled (−40 °C) extraction solvent (methanol:acetonitrile:water, 2:2:1, *v*/*v*/*v*) containing internal standards. The mixture was homogenized at 35 Hz for 4 min with steel beads using a tissue homogenizer (MM400, Retsch GmbH, Haan, Germany), followed by ice-water bath ultrasonication for 5 min. This cycle was repeated three times. Samples were then incubated at −40 °C for 1 h to precipitate proteins. Following centrifugation, 300 μL of supernatant was transferred to a 96-well filtration plate and filtered using positive pressure (6 psi for 3 min).

Chromatographic separation was performed on a Vanquish UHPLC system (Thermo Fisher Scientific Inc., Waltham, MA, USA) equipped with a Phenomenex Kinetex C18 column (2.1 mm × 50 mm, 2.6 μm, Phenomenex Inc., Torrance, CA, USA). The mobile phase consisted of (A) 0.01% acetic acid in water and (B) isopropanol:acetonitrile (1:1, *v*/*v*). Mass spectrometry detection was conducted using an Orbitrap Exploris 120 mass spectrometer Thermo Fisher Scientific, Waltham, MA, USA operating in both positive and negative electrospray ionization modes.

### 2.12. Data Analysis and Experimental Design

Experimental data were analyzed using IBM SPSS (version 28.0.1.0, IMB Corp., Armonk, NY, USA) and Microsoft Excel (version 2402, Microsoft Corp., Redmond, WA, USA. Triplicate measurements were assessed by one-way ANOVA. Results are presented as the mean ± standard deviation (SD). When ANOVA indicated significant differences (*p* < 0.05), means were compared using Duncan’s multiple range test. The experiment used a randomized complete block design within a controlled chamber calibrated to simulate a tropical climate.

For metabolomics data, multivariate statistical analysis was performed using SIMCA (version 18.0.1, Sartorius Stedim Data Analytics AB, Umeå, Sweden). Principal component analysis (PCA) was conducted as an unsupervised method, and orthogonal partial least squares discriminant analysis (OPLS-DA) as a supervised method. Differential metabolites were screened using VIP > 1 and *p* < 0.05 criteria.

## 3. Results

### 3.1. Strain Selection

The mycelial growth lengths of 20 *P. ostreatus* strains cultivated over six weeks under the SSOI method (T3 formulation) are presented in Table 2. The slowest and fastest growth lengths were recorded for strains PY9NS and PXF9, respectively (PY9NS: 00.00 ± 00 a, PXF9: 17.90 ± 0.17 i for the experimental group; PY9NS: 0.17 ± 0.29 a, PXF9: 15.67 ± 0.58 h for the control group). All other strains exhibited intermediate values. Strains sharing the same superscript letter were not statistically different, while strains with different letters differed significantly (*p* < 0.05). Based on its superior growth performance, *P. ostreatus* PXF9 was selected for all subsequent experiments.

### 3.2. Growth Rates and Transition Stages

The CSAI treatments (T1 and T2) resulted in significantly shorter colonization and initiation times compared to SSOI (T3) and NSOI (T4) treatments (*p* < 0.05; Table 3 and Table 4), underscoring the efficacy of comprehensive substrate disinfection. The duration from pinhead formation to mature fruiting body was unaffected by either disinfection method or substrate type. The shortest flush cycle was observed in T1, followed closely by T3 and T4, whereas T2 exhibited the longest cycle, indicating a substrate-dependent effect (Table 4).

### 3.3. Harvesting Parameters

The highest yield and BE were observed in T1 (3985.26 ± 2.00 ^d^ g per 24 bags and 83.03 ± 1.00 ^d^ % respectively; Table 5. T3 produced the longest stipe (7.00 ± 0.00 cm) as well as the largest stipe diameter (1.40 ± 0.10 cm) and pileus diameter (7.33 ± 0.58 cm). T2 consistently underperformed across several parameters (Table 3, Table 4 and Table 5), demonstrating the nutritional advantage of the coffee parchment and Juncao-based substrates over sawdust alone.

### 3.4. Nutritional Parameters

Most nutritional parameters varied across treatments (Table 6), with the exception of total polysaccharide, which showed no statistically significant differences among all treatments. T1 yielded the highest protein content (28.44 ± 0.52 g/100 g), which was statistically similar to T2 (27.20 ± 0.47 g/100 g). T3 exhibited low levels of protein (18.52 ± 0.21 g/100 g), carbohydrate (503.59 ± 3.44 mg/g), moisture (2.98 ± 0.26 g/100 g), and crude fiber (8.18 ± 0.27%), but demonstrated high polysaccharide (17.42 ± 1.70 mg/g), crude fat (2.76 ± 0.13 g/100 g), and ash (8.30 ± 0.39 g/100 g). T4 showed the highest carbohydrate content (586.13 ± 5.74 mg/g) alongside low polysaccharide (15.20 ± 1.65 mg/g), crude fat (1.99 ± 0.10 g/100 g), and ash content (6.54 ± 0.15 g/100 g).

### 3.5. Heavy Metals in Fruiting Bodies

The heavy metal concentrations were significantly influenced by the disinfection methods (Table 7). All tested heavy metals were consistently highest in the T3 method and lowest in T4.

### 3.6. Economic Benefits

By utilizing short or non-sterilization methods in the rural farming system, growers can cultivate *P. ostreatus* PXF9 efficiently for local markets, generating profitable income with reduced cost and effort. The current market price of oyster mushrooms in PNG is 5 USD/kg under large-scale farming conditions, where standard sterilization methods, trained professionals, and substrate costs apply. For local farmers using short or non-sterilization methods with readily available coffee parchment and *C. fugigraminus*, the only required input is spawn purchase from a commercial supplier (1 kg spawn bag costs 0.28 USD). This approach eliminates unnecessary costs associated with substrates, autoclave equipment, and inoculation and incubation chambers.

Cost inputs per method:

1. Polythene bags: 6.84 USD per 100 bags; 30 bags cost 2.05 USD.
2. Spawn: CSAI requires 300 g (0.08 USD) at 10 g per bag; SSOI and NSOI each require 750 g (0.21 USD) at 5% spawn per bag.
3. Calcium carbonate (lime-CaCO_3_): 1.43 USD per 500 g. CSAI requires 120 g (0.34 USD) at 2% of 6 kg dry weight; SSOI requires 240 g (0.68 USD) at 4% of 6 kg dry weight.
4. Quick lime (CaO): 1.21 USD per 500 g. NSOI requires 840 g (2.03 USD) at 2% of 36 L water + 2% of 6 kg dry weight.
5. Wheat bran: 2.72 USD (0.23 USD per 100 g), required in all methods.

Total costs exclude labor, steam autoclave sterilizer (CSAI only), electricity (CSAI only), and transportation to market. All primary substrates (coffee parchment and *C. fungigraminus*) are locally available at no cost.

Table 8 presents the comparative assessment of yield and profitability across three production cycles while total material costs per 30 bags is found in Supplementary Material Table S2.

### 3.7. Extracellular Enzyme Activities

Extracellular enzyme activities (laccase, manganese peroxidase, cellulase, xylanase, and alpha-amylase) were assessed at four distinct growth stages across all treatments. The complete enzyme activity profiles are presented in Supplementary Figure S7.

#### 3.7.1. Laccase Activity

Laccase activity was generally low during post-inoculation, with T4 showing the lowest activity and T1 the highest. During mycelium maturation, T2 exhibited the lowest activity (declining slightly), T4 increased moderately, and T1 remained significantly higher than all others.

During the primordia formation stage, T2 remained low, while T4 increased substantially and became significantly higher than all treatments. During the fruiting period T2 remained low, T3 declined (both statistically similar), and T4 declined sharply but remained significantly higher than the other groups (Supplementary Figure S7A).

#### 3.7.2. Manganese Peroxidase (MnP) Activity

During the post-inoculation period, T2 and T4 exhibited statistically similar low MnP activity, T3 was significantly higher, and T1 recorded the highest activity.

During the mycelium maturation, T2 declined to the lowest level. T4 increased greatly (surpassing T2 and T3 but remaining below T1), and T1 remained significantly highest.

During the primordia formation, T4 declined slightly but remained significantly higher than others.

During the fruiting period, T4 dropped sharply to the lowest level overall, while T1 maintained its previous level and remained significantly higher (Supplementary Figure S7B).

#### 3.7.3. Cellulase Activity

During the post-inoculation period, T2 and T3 were statistically similar and significantly lower than T1, which recorded the highest activity.

During the mycelium maturation, T3 maintained T2 and T4 were statistically similar (with T2 increasing slightly and T4 declining slightly), and T1 showed a sharp decline but remained significantly higher than the others.

During the primordia formation, T4 exhibited significantly higher activity than the others, with increasing activity over time. During the fruiting body period, T1 increased substantially and became significantly higher than the others (Supplementary Figure S7C).

#### 3.7.4. Xylanase Activity

During the post-inoculation, T1 was significantly lower than T3 and T4 but similar to T2; T3 and T4 were statistically similar and recorded the highest activity.

During mycelium maturation, all treatments differed significantly: T4 recorded the lowest activity and T3 the highest.

During primordia formation, all treatments displayed high xylanase activity and were statistically similar (Supplementary Figure S7D).

#### 3.7.5. Alpha-Amylase Activity

During the post-inoculation, T1 and T2 exhibited very low (statistically similar) while T4 displayed the highest activity.

During the mycelium maturation, activity was generally high and did not differ significantly among treatments.

During the primordia formation period, T3 and T4 were statistically similar and exhibited the highest activity.

During the fruiting stage, T2, T3, and T4 were statistically similar, whereas T1 increased slightly and became significantly higher than the others (Supplementary Figure S7E).

### 3.8. Metabolomic Profiling

Untargeted LC-MS metabolomics analysis detected 46,898 features, of which 1591 substances achieved secondary identification. Identified metabolite classes included acylcarnitines (involved in fatty acid transport and energy metabolism), phytochemicals (naringenin, [4]-gingerdiol, 6-methylgingediol), lipids and derivatives (LPE, phytosphingosine, phytoprostanes), alkaloids (tryptamine, stachydrine), and terpenes (ergosterol peroxide, alpha-bisabolol, labdane derivatives).

**Figure 1:**
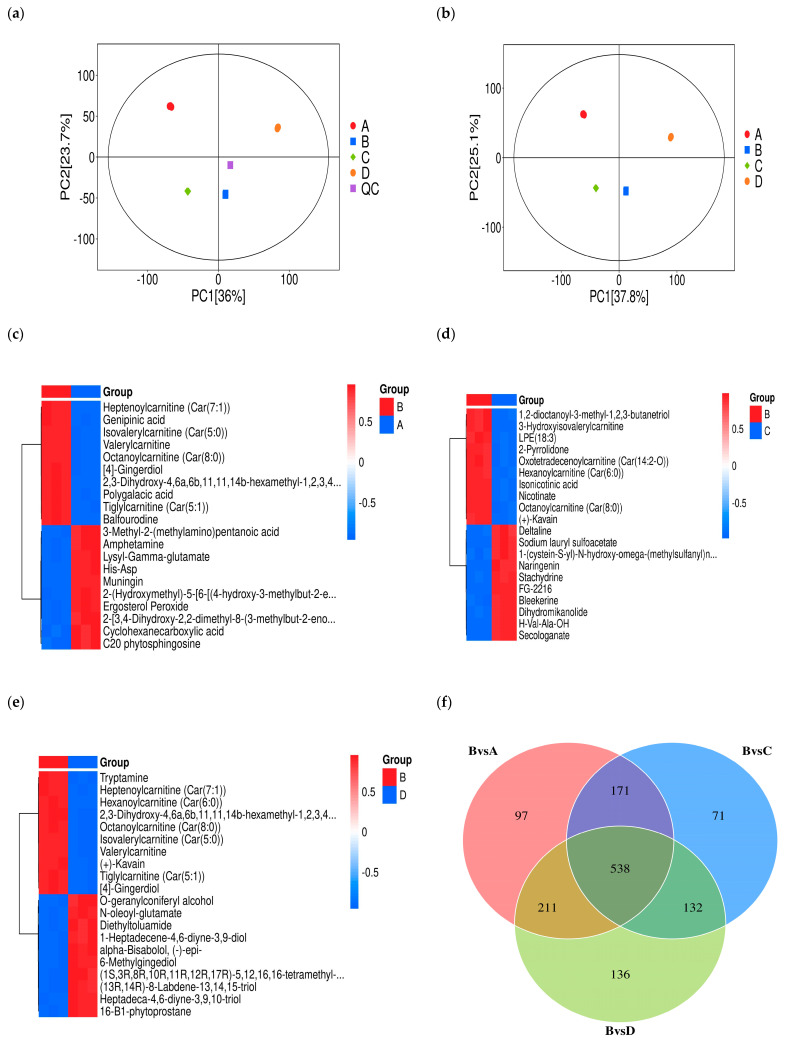
PCA score plot showing clear metabolic separation among groups. (**a**) all experimental samples including QC sample (A = T1, B = T2, C = T3, D = T4, QC = Quality control) (*n* = 15), (**b**) only experimental samples (*n* = 12). (**c**) Hierarchical clustering heatmap of B vs. A (T2 vs. T1). (**d**) Hierarchical clustering heatmap of B vs. C (T2 vs. T3). (**e**) Hierarchical clustering heatmap of B vs. D (T2 vs. T4). (**f**) Venn diagram showing overlap of differential metabolites across all pairwise comparisons. A total of 46,898 features were detected, of which 1591 substances achieved secondary identification. Metabolite classes included acylcarnitines, phytochemicals (naringenin, [4]-gingerdiol, 6-methylgingediol), lipids and derivatives, alkaloids (tryptamine, stachydrine), and terpenes (ergosterol peroxide, alpha-bisabolol, labdane derivatives).

Principal component analysis (PCA) and orthogonal partial least squares discriminant analysis (OPLS-DA) models revealed clear separation among the four treatment groups. PC1 and PC2 explained 59.7% of the total variance, and the OPLS-DA models achieved perfect discrimination (Q^2^ > 0.98, R^2^Y = 1.0) (Figure 1a,b and Supplementary Table S3 respectively).

Hierarchical clustering heatmaps display the top 20 up- and down-regulated metabolites across treatment groups (A = T1, B = T2, C = T3, D = T4) relative to the control (B, T2 revealing limited similarities in metabolite profiles among groups (Figure 1c–e). A Venn diagram identifies a core set of 538 metabolites commonly altered in all pairwise comparisons (Figure 1f). T4 (NSOI) exhibited the highest number of unique differential metabolites (136), enriched in acylcarnitines, alkaloids (tryptamine), phytochemicals ((+)-kavain, [4]-gingerdiol), and terpenoids. T1 (CSAI experimental) contained 97 unique metabolites, characterized by acylcarnitines and energy-related compounds. T3 (SSOI) had 71 unique metabolites associated with hemicellulose-derived compounds.

## 4. Discussion

### 4.1. Growth Transition and Harvesting Parameters

Mycelial growth, developmental transitions, and yield parameters were significantly influenced by the disinfection methods and substrates used (*p* ≤ 0.05). The achievement of complete colonization within 21 days (T1 and T2), combined with high yield and BE (T1: 3985.26 ± 2.00 g and 83.03 ± 1.00%, respectively), as well as a shorter flush cycle (T1: 7.33 ± 0.58 days) in the CSAI treatment is primarily attributable to the complete elimination of competing and contaminating microorganisms, followed by aseptic spawn inoculation [6]. In contrast, the 28-day colonization period observed in the short sterilization open inoculation (SSOI) and non-sterilization open inoculation (NSOI) treatments, along with their respective average yields (3091.69 ± 3.00 g and 2592.30 ± 3.00 g, respectively) and BE values (64.41 ± 1.00% and 54.05 ± 1.00%), may be attributed to the presence of competing microorganisms. Additionally, excessive lime concentration may represent another contributing factor to the prolonged colonization periods and reduced yields in these treatments.

A previous study by Zhang et al. [8], using an approach similar to our T4 treatment, reported complete substrate colonization within 21 days and a biological efficiency (BE) of 112.78%. Although the substrate was comparable, their protocol incorporated corn powder as an additive with only 2% lime, whereas the present study used wheat bran and 4% lime. This comparison suggests that appropriate supplementation with lime and corn flour under non-sterile cultivation conditions may enhance mycelial growth [8]. Supporting this, Ghareeb [42] demonstrated that elevated lime concentrations negatively affected growth and delayed colonization, observations also evident in our SSOI and NSOI treatments. These findings align with those of Obodai et al. [43], who reported mycelial completion times ranging from 15 to 33 days. Furthermore, the results of our CSAI treatment are consistent with the 21-day colonization period documented by Orngu et al. [44] for sawdust-based substrates amended with cow and pig dung after 15 min of sterilization.

The BE values obtained in this study align with, exceed, or approximate previously reported findings. Dissasa [45] reported values ranging from 14.44% to 61.92%, while Fan et al. [46] recorded 96.5%, both using coffee husk as substrate. Furthermore, our BE values are consistent with and fall between those reported by Shrestha et al. [47], who obtained the highest BE under sterilization conditions (101.38%), followed by hot water sterilization (90.39%) and chemical sterilization (79.75%) using sawdust and wheat straw. Similarly, the duration from initiation to primordia formation in our CSAI treatment corresponds with their reported range of 8 to 9 days for complete substrate colonization [48]. The interval between inoculation and pinhead formation (30 to 40 days) aligns with the findings of Obodai et al. [43], who documented 19 to 38 days when evaluating eight lignocellulosic byproducts as substrates for *P. ostreatus*. In contrast, Subedi et al. [48] reported a shorter range of 13 to 20 days using paddy straw and water hyacinth, underscoring the influence of substrate selection. The achievement of high harvesting parameter values in our experiment may be attributed to a combination of disinfection methods, substrate selection, fungal strain, and environmental factors [49,50].

Pileus diameter in the present study ranged from 5.25 ± 0.17 cm to 7.33 ± 0.58 cm, which aligns with previously reported values of 4.64–7.28 cm [12] and 4.63–5.21 cm [48], indicating normal and consistent fruiting body development. Stipe length ranged from 4.83 ± 0.21 cm to 7.00 ± 0.00 cm, whereas earlier studies reported values of 2.81–3.92 cm [12], 2.50–3.44 cm [48], and 4.3 ± 0.9 cm to 7.1 ± 1.4 cm when Juncao-based substrates were used [4]. This variation highlights the influence of Juncao substrates on fruiting body morphology, likely attributable to their high nutritional content, including nitrogen and carbon, which are known to affect fruiting body development [13]. Based on the comparable outcomes observed, the methods used in this study are strongly recommended for adoption by both small- and large-scale farmers using locally available substrates.

The reduced performance of T5 (attributed to strain selection growth rate) and T2 across several harvesting parameters is attributable to the low protein content of sawdust, which is insufficient to support fungal growth [12,51]. This observation is supported by a comparative nutritional analysis conducted by Prof. Lin Zhanxi, which reported 1.19% protein in sawdust versus 5.91% in *C. fungigraminus* [13], underscoring the critical role of substrate selection in mushroom cultivation. Furthermore, the extended interval between flushes observed in T2 (12 days) may be due to the high lignocellulosic content of sawdust. Previous studies have reported that substrates rich in lignin and cellulose tend to prolong harvesting cycles [52]. Although the flush intervals recorded in this study fall within the range of 6.33–16.72 days documented by Bhatti et al. [53] and Obodai et al. [43], a shorter interval remained the target.

### 4.2. Nutrient Content

Protein content was strongly influenced by the disinfection method, with CSAI treatment yielding the highest and statistically significant values of 28.44 ± 0.52 g/100 g (T1) and 27.20 ± 0.47 g/100 g (T2). These results exceed those reported by Aimable et al. [5] and Claude et al. [4], who also utilized Juncao-based substrate, thereby highlighting the importance of supplementing coffee parchment with Juncao substrates and selecting an appropriate *P. ostreatus* strain. Nevertheless, these findings are consistent with those of Harikrishna et al. [54], who reported values ranging from 22.75 to 26.83 g/100 g, which approximate the upper range of our results. Essien et al. [55] documented values of 21.71 ± 1.09 g/100 g and 22.45 ± 1.15 g/100 g for the first and final harvests, respectively, using shade drying, indicating that both flush cycles and drying method contribute to nutritional variation. In contrast, Kajendran et al. [56] reported a higher value of 30.0 g/100 g using freeze drying, which more closely aligns with our highest recorded value, further demonstrating that drying significantly influences nutrient content.

All carbohydrate contents surpassed previously reported values, with T4 recording the highest (586.13 ± 5.74 mg/g). Essien et al. [55] and Kajendran et al. [56] reported values of 0.45 mg/g and 497.5 mg/g, respectively. Furthermore, Harikrishna et al. [54] documented lower values ranging from 412.67 to 456.00 mg/g. Total polysaccharide content was not significantly influenced by either substrate type or disinfection methods. The values obtained in the present study (15.20 ± 1.65 a for T4 to 17.42 ± 1.70 a mg/g for T3) were lower than those reported by Claude et al. [4], who observed a range of 23.1 ± 0.4 mg/g to 27.7 ± 0.5 mg/g. Collectively, these findings suggest that both carbohydrate and polysaccharide content are strongly influenced by the cultivated strain [49].

The moisture and fat contents recorded in this study were lower than those reported by Essien et al. [55] (11.5 g/100 g and 4.52–4.77 g/100 g, respectively) and Kajendran et al. [56] (12.27 g/100 g and 0.63 g/100 g, respectively) but were higher than their respective fiber findings of 4.6% and 0.11%, whereas our values ranged from 8.18 ± 0.27% to 9.87 ± 0.22%. According to Yu et al. [57], mushroom dietary fiber functions as a prebiotic, promoting beneficial gut microbiota and the production of short-chain fatty acids, thereby supporting gut barrier function and metabolic health. Consequently, consumption of mushrooms cultivated using the methods described herein may confer benefits to gut health. This notion is further supported by Caz et al. [58], who reported that the dietary fiber component of *P. ostreatus* may help reduce triglyceride accumulation in the liver. The ash content observed in this study aligns well with both Essien et al. [55] and Kajendran et al. [56], while surpassing that reported by Aimable et al. [5]. The consistent, comparable, and in some cases superior nutritional values obtained related to previous studies highlight the robustness and validity of the methodological approach applied here. Additionally, variation in nutritional content is clearly influenced by the fruiting body drying method, environmental factors, selected strain, lime concentration, and, most importantly, the disinfection methods [49,50,54].

### 4.3. Extracellular Enzyme Activities

The enzyme activities characterized in this study demonstrate that disinfection methods influence both the timing and magnitude of laccase, manganese peroxidase (MnP), cellulase, xylanase, and alpha-amylase activity. Laccase activity exhibited significant differences during the first three cultivation periods, indicating strong dependence on substrate composition and disinfection methods. Previous studies have reported that substrate selection and disinfection methods greatly influence enzymatic activity, in addition to environmental factors, pH, and fungal strain [16]. Our findings correspond with those of Claude et al. [4], who observed low laccase activity during the early cultivation period followed by an increase during mid-cultivation and a subsequent decline during fruiting. Furthermore, Aimable et al. [5] confirmed the variation in laccase activity rates across different growth cycles.

MnP activity peaked during the post-inoculation and mycelial maturation periods, then declined through primordia and fruiting stages. The elevated MnP activity observed from post-inoculation through primordia formation demonstrates its essential role in lignin degradation within the substrates [59]. This finding is supported by those of Claude et al. [4] and Aimable et al. [5], who also reported high MnP activity during the incubation period followed by a decrease during the fruiting stage.

Cellulase activity gradually increased, reaching peak levels during primordia and fruiting stages, indicating its significant role during transformative phases. This finding is consistent with that of Aimable et al. [5], who also reported a gradual increase in cellulase activity throughout the incubation period, with maximum activity observed from the primordia through the fruiting stage.

Xylanase activity remained high across all treatments throughout the entire cultivation period, indicating continuous hemicellulose degradation [60]. Our findings are consistent with those of Aimable et al. [5] but differ from those of Claude et al. [4], who reported very low xylanase activity.

Alpha-amylase activity gradually increased from post-inoculation, peaked from mycelial maturation through primordia formation, and subsequently declined during the fruiting stage. These findings corroborate the observation of Inácio et al. [61], who described alpha-amylase as relatively low during the initial mycelial colonization, with a gradual increase as the mycelium colonizes the substrate.

### 4.4. Metabolomic Profiles and Their Integration with Enzyme Activity

Multivariate analysis revealed clear separation among the four treatment groups, confirming distinct metabolomic fingerprints for each disinfection method. A core set of 538 metabolites was commonly altered across all pairwise comparisons, representing a conserved metabolic response involved in amino acid, carbohydrate, and cofactor metabolism (Figure 1). Each treatment exhibited unique metabolic signatures aligned with enzyme activity patterns.

T1 (CSAI) contained 97 unique metabolites, predominantly acylcarnitines (fatty acid transporters for mitochondrial β-oxidation [62]), which may indicate active energy metabolism from cellulose-derived glucose. This aligns with high cellulase activity, supporting efficient substrate degradation and the highest yield. The absence of stress-related metabolites (enriched in T4) may suggest that sterilization minimizes metabolic stress, though targeted validation would be required to confirm this interpretation. T2 (CSAI control) displayed low enzyme activities and the fewest unique metabolites, reflecting the nutritional inferiority of sawdust alone. T3 (SSOI) contained 71 unique hemicellulose-derived metabolites, reflecting partial hemicellulose breakdown under short sterilization that releases fermentable sugars without strong stress responses; high xylanase activity aligns with this signature. T4 (NSOI) exhibited the highest number of unique metabolites (136), enriched in acylcarnitines, alkaloids (tryptamine), phytochemicals, and terpenoids. This profile is consistent with metabolic stress under non-sterile conditions, potentially involving: (1) enhanced energy metabolism (acylcarnitines); (2) nitrogen reallocation (tryptamine); and (3) antioxidant production (phytochemicals). These adjustments are consistent with high laccase activity during primordia formation, as laccase mediates phenolic compound release. Terpenoid enrichment (ergosterol peroxide, alpha-bisabolol) further supports stress adaptation [63]. Acylcarnitines (elevated in T1/T4) facilitate β-oxidation [62]; phytochemicals like naringenin possess antioxidant properties; alkaloids (highest in T4) influence stress adaptation; terpenes (highest in T1) exhibit antimicrobial/antioxidant activities [63].

Sterilization (T1) may promote efficient energy metabolism and high yield potentially through cellulase activity and acylcarnitine-driven energy production, though further validation is needed. Non-sterilization (T4) appears to impose metabolic stress, which could trigger laccase-mediated phenolic release and accumulation of alkaloids and terpenoids. Short sterilization (T3) occupies an intermediate position, likely involving xylanase-driven hemicellulose degradation. These findings provide molecular insight into how disinfection methods affect mushroom physiology, nutritional quality, and stress adaptation.

### 4.5. Heavy Metals

The observed differences in heavy metal concentrations in the fruiting bodies remained within or below the consumable limits. According to Yongning Wu [64], the international food safety standards for arsenic, lead, mercury, and cadmium are 0.1–0.2 mg/kg, 0.01–3 mg/kg, 0.5–1 mg/kg, and 0.05–2 mg/kg, respectively. These results affirm the suitability of the cultivation methods for both health standards and marketability. The slightly elevated values observed in T3 compared to other treatments may be attributed to the partial sterilization method, which potentially reduced microbial competition without fully eliminating metal-mobilizing microorganisms, thereby facilitating increased metal uptake. Conversely, the lowest concentrations recorded in T4 suggest that lime treatment may promote metal complexation or precipitation, thereby reducing bioavailability. Collectively, these findings underscore the importance of disinfection method selection not only for yield optimization but also for ensuring food safety.

### 4.6. Economic Benefits

In addition to the biological and nutritional advantages, the economic viability of each disinfection method is a critical factor for adoption by small-scale farmers. A cost–benefit analysis was conducted based on the current market price of oyster mushrooms in PNG (5 USD/kg) and the material costs incurred for each treatment (Table 8 and Supplementary Table S3). For local farmers using short or non-sterilization methods with locally available coffee parchment and Giant Juncao grass, the primary recurring cost is spawn purchase, as the main substrates are obtainable at no cost.

Total material costs per 30 bags were 5.19 USD for CSAI, 5.66 USD for SSOI, and 7.01 USD for NSOI. The higher cost for NSOI was primarily attributable to the greater quantity of lime required. Despite these differences, all three methods generated profits after three harvest cycles: CSAI T1 yielded the highest profit (14.76 USD per 24 bags), followed by SSOI (9.88 USD), CSAI T2 (6.56 USD), and NSOI (5.96 USD). Although CSAI T1 maximized returns, SSOI and NSOI remained economically viable options for resource-limited farmers, requiring lower initial investment in equipment such as autoclaves and specialized inoculation chambers. These results demonstrate that even with minimal inputs, farmers can achieve profitable returns by utilizing locally available waste materials.

### 4.7. Limitations and Future Directions

This study has several limitations. First, the experiments were conducted under controlled chamber conditions designed to reflect the tropical climate of the Highlands Region of PNG and may not fully represent the variability of outdoor or smallholder farming environments in other regions. Second, further optimization of lime concentration and formulation adjustments for the SSOI and NSOI methods may improve yields and reduce colonization times. Third, the economic analysis focused on material costs; a full life-cycle assessment incorporating labor, energy, and equipment depreciation would provide a more comprehensive understanding of profitability for farmers with varying resource levels.

Future research should include long-term field trials under diverse environmental conditions to validate scalability. In particular, pilot-scale trials involving 100–500 bags per treatment would help assess the feasibility of SSOI and NSOI methods under semi-commercial conditions. Additionally, industrial-scale validation should evaluate substrate handling efficiency, contamination risks, and labor costs across multiple cultivation cycles. Exploring alternative low-cost additives (e.g., locally available agricultural byproducts) to enhance the performance of non-sterilization methods is also recommended. Finally, collaborations with farmer cooperatives in PNG would facilitate technology transfer and provide real-world feedback on method adoption and economic returns.

## 5. Conclusions

This study successfully identified *P. ostreatus* PXF9 as the superior strain for cultivation on coffee parchment and *C. fungigraminus* substrates. Among the disinfection methods evaluated, comprehensive steam autoclave sterilization (CSAI, T1) delivered the highest performance in terms of yield (3985 g/24 bags), biological efficiency (83.03%), protein content (28.4%), and profit (14.76 USD), with the fastest colonization time (21 days). However, for resource-limited farmers, short-period sterilization (SSOI, T3) and lime-based non-sterilization (NSOI, T4) remain economically viable, generating profits of 9.88 USD and 5.96 USD per 24 bags, respectively, without requiring autoclave equipment. Notably, T4 produced the lowest heavy metal concentrations, all of which remained within safe limits, while T3 yielded the largest fruiting bodies.

Enzyme activity profiling revealed method-specific patterns: laccase and manganese peroxidase (MnP) peaked during colonization, cellulase increased during fruiting, xylanase remained consistently elevated throughout cultivation, and alpha-amylase peaked during active mycelium growth. Metabolomic profiling confirmed clear separation among treatments, with T4 yielding the highest diversity of unique metabolites (136) and T1 displaying enhanced markers of energy metabolism. Key bioactive compounds, including naringenin and ergosterol peroxide, were detected across all treatments, confirming the functional food potential of the cultivated mushrooms.

Practical recommendations are as follows: CSAI is recommended for maximum returns, SSOI as a balanced low-technology option, and NSOI where food safety is prioritized. All nutritional parameters aligned with or exceeded literature values, and heavy metal concentrations remained within international safety limits. These findings provide farmers in PNG with flexible, evidence-based options for sustainable mushroom cultivation using locally available agricultural wastes, supporting both food security and income generation in resource-limited tropical regions.

## Figures and Tables

**Table 1:** Substrate formulations for different cultivation methods.

Cultivation Group	*C. fungigraminus* (%)	Coffee Parchment (%)	Sawdust (%)	Wheat Bran (%)	Lime Mix Together (%)	Lime Added After (%)	Lime for Soaking (%)	Total (%)
T1	48	30	-	20	2	-	-	100
T2			78	20	2	-	-	100
T3	48	30	-	20	2	2	-	102
T4	48	30	-	20	2	-	2	102
T5	-	-	78	20	2	2	-	102

Calcium carbonate (CaCO_3_) was used as the lime source for T1, T2, T3, and T5, while calcium oxide (CaO) was used for treatment T4.

**Table 2:** Growth rate of 20 *P. ostreatus* varieties from week 1 to 6 under the SSOI method.

Number	*P. ostreatus* Strain Codes	Experimental Mycelium Growth Length (cm)	Control Mycelium Growth Length (cm)
1	PXt	3.10 ± 0.17 ^b^	0.67 ± 0.29 ^de^
2	P969	7.17 ± 1.04 ^de^	8.17 ± 1.61 ^de^
3	PDHP	3.23 ± 0.40 ^b^	1.00 ± 0.50 ^ab^
4	PXF9	17.90 ± 0.17 ^i^	15.67 ± 0.58 ^h^
5	PXF2	11.07 ± 1.01 ^f^	15.67 ± 0.58 ^h^
6	PYH8	6.67 ± 0.65 ^d^	1.67 ± 1.04 ^ab^
7	PXHP	16.83 ± 0.76 ^hi^	10.00 ± 2.00 ^fg^
8	PtP2	7.97 ± 0.42 ^e^	2.00 ± 1.00 ^b^
9	P615	6.10 ± 0.53 ^cd^	4.00 ± 1.00 ^c^
10	P246	16.33 ± 0.58 ^h^	10.33 ± 0.58 ^fg^
11	PTCHP	5.33 ± 1.04 ^c^	4.83 ± 0.76 ^c^
12	PX50	7.17 ± 1.04 ^de^	14.57 ± 1.12 ^h^
13	P313	8.00 ± 1.00 ^e^	6.83 ± 1.17 ^d^
14	PY9NS	00.00 ± 00 ^a^	0.17 ± 0.29 ^a^
15	P7448	14.53 ± 0.95 ^g^	9.57 ± 0.71 ^ef^
16	PFP	11.33 ± 0.76 ^f^	11.50 ± 1.32 ^g^
17	PJPg	10.17 ± 0.29 ^f^	8.00 ± 0.60 ^de^
18	PZQ615	0.50 ± 0.50 ^a^	1.17 ± 0.76 ^ab^
19	PXF11	8.100 ± 0.46 ^e^	15.83 ± 0.29 ^h^
20	PX25	0.33 ± 0.29 ^a^	0.23 ± 0.40 ^a^

Different letters superscripted next to the values under the experimental and control columns represent significant differences at the 0.05 level according to Duncan’s test.

**Table 3:** Mycelium colonization rate of the different cultivation groups.

Cultivation Group	Week 1 (cm)	Week 2 (cm)	Week 3 (cm)	Week 4 (cm)	Complete Mycelium Run Days
T1	1.28 ± 0.11 ^a^	4.51 ± 0.13 ^c^	2.61 ± 0.40 ^b^	completed	21.33 ± 0.58 ^a^
T2	1.02 ± 0.80 ^a^	4.32 ± 0.18 ^c^	1.86 ± 0.05 ^a^	completed	21.33 ± 0.58 ^a^
T3	2.67 ± 0.29 ^c^	3.25 ± 0.43 ^b^	2.17 ± 0.14 ^ab^	1.83 ± 0.52 ^a^	28.00 ± 1.00 ^b^
T4	1.99 ± 0.32 ^b^	2.65 ± 0.16 ^a^	2.41 ± 0.53 ^ab^	2.53 ± 0.12 ^b^	28.00 ± 1.00 ^b^

Data are presented as mean ± standard deviation (SD). Different superscript letters (a–c) within the same column denote significant differences at *p* ≤ 0.05 based on Duncan’s multiple range test. Treatment codes: T1, CSAI (experimental); T2, CSAI (control); T3, SSOI (experimental); T4, NSOI.

**Table 4:** Growth and transition stages.

Cultivation Group	Initiation to Pinhead Formation (Days)	Inoculation to Pinhead Formation (Days)	Pinhead to Mature Fruiting Bodies (Days)	Period Between Flushes (Days)
T1	9.00 ± 1.00 ^a^	30.00 ± 1.00 ^a^	4.33 ± 0.58 ^a^	7.33 ± 0.58 ^a^
T2	9.33 ± 0.57 ^a^	30.33 ± 0.58 ^a^	4.67 ± 0.58 ^a^	12.00 ± 1.00 ^c^
T3	10.00 ± 1.00 ^a^	38.00 ± 1.00 ^b^	4.67 ± 0.58 ^a^	8.00 ± 1.00 ^a^
T4	13.00 ± 1.00 ^b^	40.67 ± 0.58 ^c^	4.33 ± 0.58 ^a^	8.33 ± 1.53 ^b^

Data are presented as mean ± standard deviation (SD). Different superscript letters (a–c) within the same column indicate significant differences at *p* ≤ 0.05 according to Duncan’s multiple range test. Treatment codes: T1, CSAI (experimental); T2, CSAI (control); T3, SSOI (experimental); T4, NSOI.

**Table 5:** Quantitative yield and morphological parameters of PXF9 from the fruiting body based on different treatment groups.

Cultivation Group	Yield After 3 Flushes (g)	Stipe Length (cm)	Stipe Diameter (cm)	Pilus Diameter (cm)	Biological Efficiency (%)
T1	3985.26 ± 2.00 ^d^	6.23 ± 0.68 ^b^	1.15 ± 0.17 ^a^	5.67 ± 1.15 ^a^	83.03 ± 1.00 ^d^
T2	2354.20 ± 2.00 ^a^	4.83 ± 0.21 ^a^	0.99 ± 0.01 ^a^	5.25 ± 0.17 ^a^	49.05 ± 1.00 ^a^
T3	3091.69 ± 3.00 ^c^	7.00 ± 0.00 ^b^	1.40 ± 0.10 ^b^	7.33 ± 0.58 ^b^	64.41 ± 1.00 ^c^
T4	2592.30 ± 3.00 ^b^	6.17 ± 0.76 ^b^	1.08 ± 0.12 ^a^	6.13 ± 0.81 ^a^	54.05 ± 1.00 ^b^

Data are presented as mean ± standard deviation (SD). Different superscripts (a–d) in the same column indicate significant differences at the 0.05 level (*p* ≤ 0.05) according to Duncan’s multiple range test. T1: CSAI experimental; T2: CSAI control; T3: SSOI experimental; T4: NSOI.

**Table 6:** Nutrient content of mushrooms from different disinfection methods.

Cultivation Group	Crude Protein (g/100 g)	Carbohydrate (mg/g Dry Weight)	Total Polysaccharide (mg/g Dry Weight)	Moisture Content (g/100 g)	Crude Fat (g/100 g)	Crude Fire (%)	Total Ash (g/100 g)
T1	28.44 ± 0.52 ^c^	526.56 ± 7.06 ^b^	17.02 ± 2.90 ^a^	7.25 ± 0.16 ^d^	2.53 ± 0.14 ^c^	8.52 ± 0.34 ^ab^	7.91 ± 0.28 ^b^
T2	27.20 ± 0.47 ^c^	558.27 ± 6.32 ^c^	15.86 ± 1.06 ^a^	6.60 ± 0.06 ^c^	2.22 ± 0.11 ^b^	9.06 ± 0.04 ^b^	6.99 ± 0.05 ^a^
T3	18.52 ± 0.21 ^a^	503.59 ± 3.44 ^a^	17.42 ± 1.70 ^a^	2.98 ± 0.26 ^a^	2.76 ± 0.13 ^d^	8.18 ± 0.27 ^a^	8.30 ± 0.39 ^b^
T4	20.04 ± 0.36 ^b^	586.13 ± 5.74 ^d^	15.20 ± 1.65 ^a^	6.00 ± 0.17 ^b^	1.99 ± 0.10 ^a^	9.87 ± 0.22 ^c^	6.54 ± 0.15 ^a^

Data are presented as mean ± standard deviation (SD). Different superscript letters (a–d) within the same column indicate significant differences at *p* ≤ 0.05 according to Duncan’s multiple range test. Treatment codes: T1, CSAI (experimental); T2, CSAI (control); T3, SSOI (experimental); T4, NSOI.

**Table 7:** Heavy metal concentrations based on the disinfection method.

Cultivation Group	Arsenic (mg/kg)	Lead (mg/kg)	Mercury (mg/kg)	Cadmium (mg/kg)
T1	0.17 ± 0.00 ^c^	0.08 ± 0.00 ^c^	0.32 ± 0.01 ^c^	0.03 ± 0.01 ^bc^
T2	0.14 ± 0.00 ^b^	0.07 ± 0.00 ^b^	0.29 ± 0.2 ^b^	0.02 ± 0.00 ^ab^
T3	0.18 ± 0.01 ^d^	0.09 ± 0.01 ^d^	0.36 ± 0.01 ^d^	0.03 ± 0.00 ^c^
T4	0.13 ± 0.01 ^a^	0.06 ± 0.00 ^a^	0.25 ± 0.01 ^a^	0.02 ± 0.01 ^a^

Data are presented as mean ± standard deviation (SD). Different superscript letters (a–d) within the same column indicate significant differences at *p* ≤ 0.05 according to Duncan’s multiple range test. Treatment codes: T1, CSAI (experimental); T2, CSAI (control); T3, SSOI (experimental); T4, NSOI.

**Table 8:** Comparative assessment of yield and profitability across cultivation groups over three production cycles.

Cultivation Groups	Yield (kg per 24 Bags) of 3 Cycles	Gross Income (USD)	Profit (USD)
CSAI T1	3.99	19.95	19.95 − 5.19 = 14.76
CSAI T2	2.35	11.75	11.75 − 5.19 = 6.56
SSOI T3	3.09	15.45	15.45 − 5.66 = 9.88
NSOI T4	2.59	12.95	12.95 − 7.01 = 5.96

These results demonstrate that while CSAI (T1) yields the highest profit, SSOI and NSOI remain economically viable options for resource-limited farmers, requiring lower initial investment in equipment and infrastructure.

## Data Availability

The data corresponding to this research will be made available upon genuine request.

## Supplementary Materials

The following supporting information can be downloaded at: https://www.mdpi.com/article/10.3390/jof12060432/s1, Figure S1. A: Grinding of fresh *C. fungigraminus*, B: *C. fungigraminus* dried using sun light, C: dry coffee husk and D; dry sawdust. Figure S2. A: sliced potato in boiler, B: collection of effluent and C: PDA collected in test tube ready to be sterilized at 121 °C for 20 min. Figure S3. A: Substrate added in a mixer, B: sawdust soaked in water for moisture absorption, and C: substrate mixing in a mixer. Figure S4. A: 15 min set for pasteurization, B: substrate in polythene bags after pasteurization, C: substrate mixed with 2% lime, D: palm squeeze test for moisture content check, E: inoculated test tubes for strain selection, and F: 5% spawn inoculation to 500 g substrate in polythene bags. Figure S5. Stepwise procedure for NSOI preparation and inoculation. A: Substrate soaked in 2% lime water, B: squeezing out of excess water, C: mixing and addition of 2% lime, D: packing and weighing of 500 g substrate, E; weighing of 5% spawn, and F; inoculation. Figure S6. Mycelium growth to fruiting and harvesting stages. A, 2 weeks mycelium run for T1; B, 2 weeks mycelium run for T2; C, 4 weeks mycelium run for T3; D, 4 weeks mycelium run for T4; E, fruiting stage for T1; F, fruiting stage for T2; G, fruiting stage for T3; H, fruiting stage for T4; I, weighing of fruiting bodies (similar for all harvested treatments); J, measuring of physical structure (similar for all harvested treatments). Figure S7. Comprehensive visualization of extracellular enzyme activities and metabolomic profiles of *P. ostreatus* PXF9 across different disinfection treatments. (A) Laccase activity; (B) manganese peroxidase (MnP) activity; (C) cellulase activity; (D) xylanase activity; (E) alpha-amylase activity. Enzyme activities were assessed at four growth stages: post-inoculation (PI), mycelium maturation (MM), primordia formation (PF), and fruiting body (FB). Different letters above bars indicate significant differences at *p* < 0.05 according to Duncan’s multiple range test. Treatment codes: T1, CSAI (experimental); T2, CSAI (control); T3, SSOI; T4, NSOI. Table S1. Composition of Potato Dextrose Agar (PDA). Table S2. OPLS-DA Model Parameters and Validation. Table S3. Total material cost per 30 bags.
