# Supplementary material for: Oyster Mushroom Cultivation on Coffee Parchment and Cenchrus fungigraminus: A Comparison of Disinfection Methods
Source: J Fungi (Basel). 2026 Jun 12;12(6):432. doi: 10.3390/jof12060432 (PMC13302646; doi:10.3390/jof12060432)
Supplement: Supplementary file 1 [file jof-12-00432-s001.zip › jof-4309689-Supplymentry.pdf]

Supplementary Figures

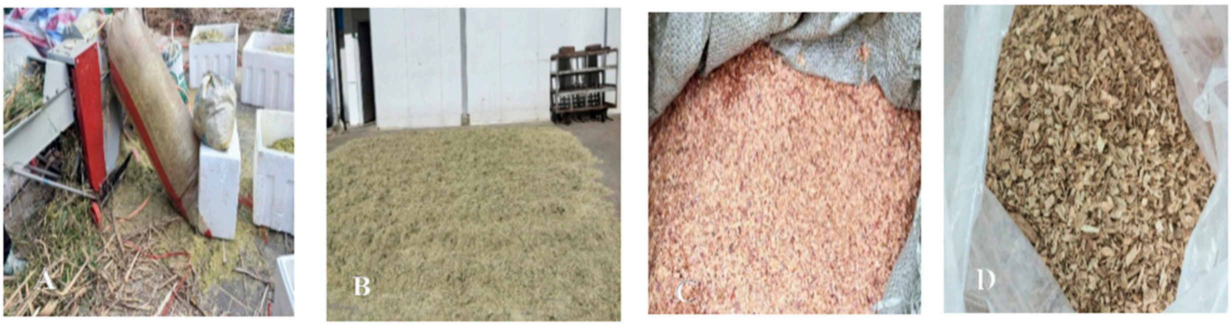

Figure S1. A; Grinding of fresh *C.fungigraminus,* B; *C.fungigraminus* dried using sun light, C; dry coffee husk and D; dry sawdust

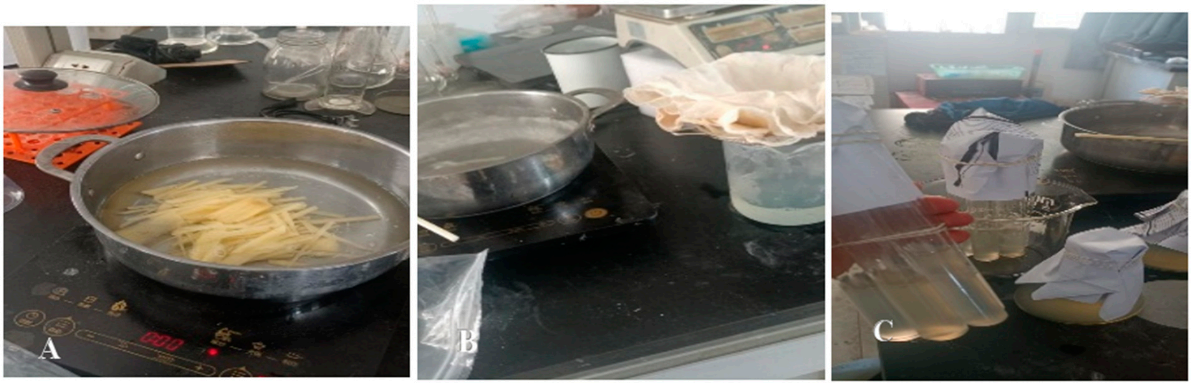

Figure S2. A; sliced potato in boiler, B; collection of effluent and C; PDA collected in test tube ready to be sterilized at 121 °C for 20 minutes.

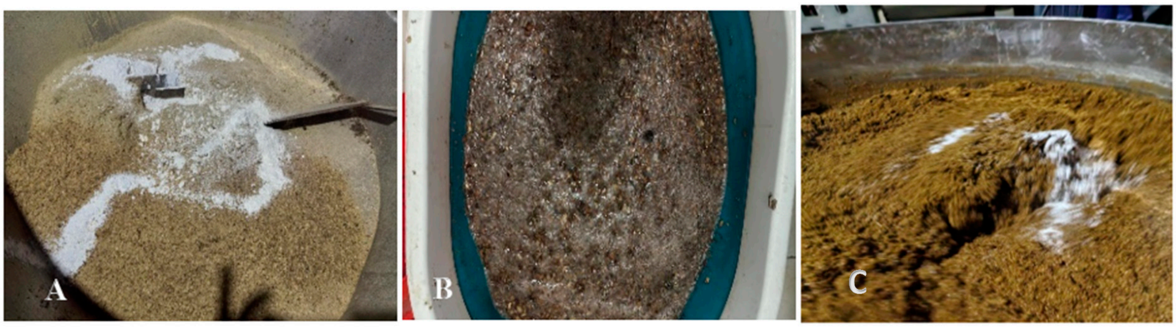

Figure S3. A; substrate added in a mixer, B; sawdust soaked in water for moisture absorption, and C; substrate mixing in a mixer

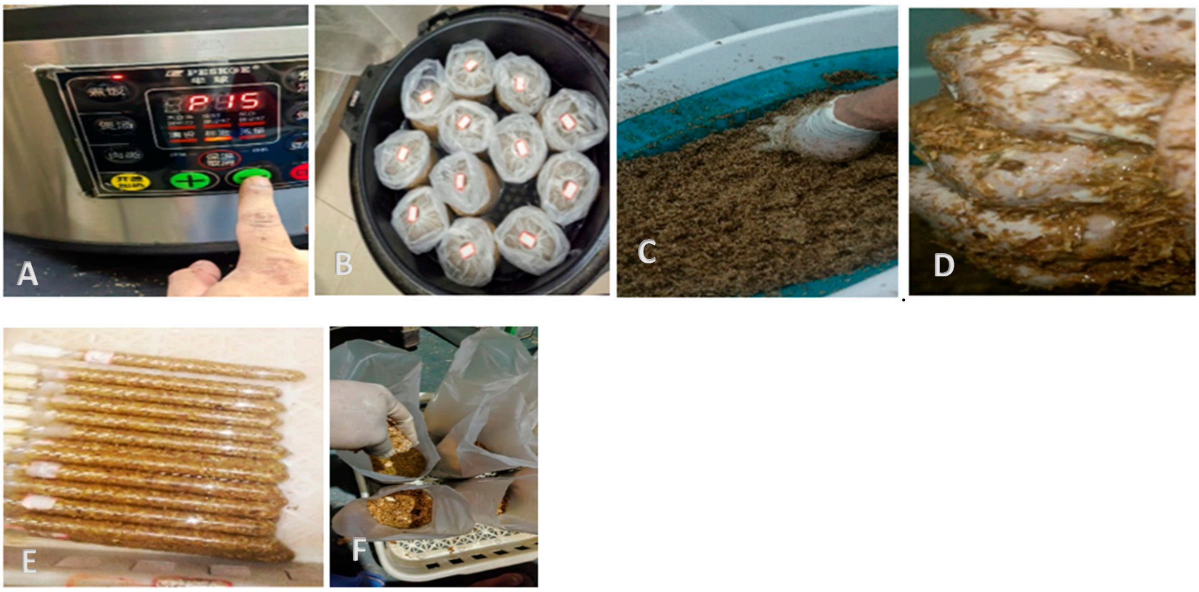

Figure S4. A;15 min set for pasteurization, B; substrate in polythene bags after pasteurization, C; substrate mixed with 2% lime, D; palm squeeze test for moisture content check, E; inoculated test tubes for strain selection, and F; 5% spawn inoculation to 500 g substrate in polythene bags.

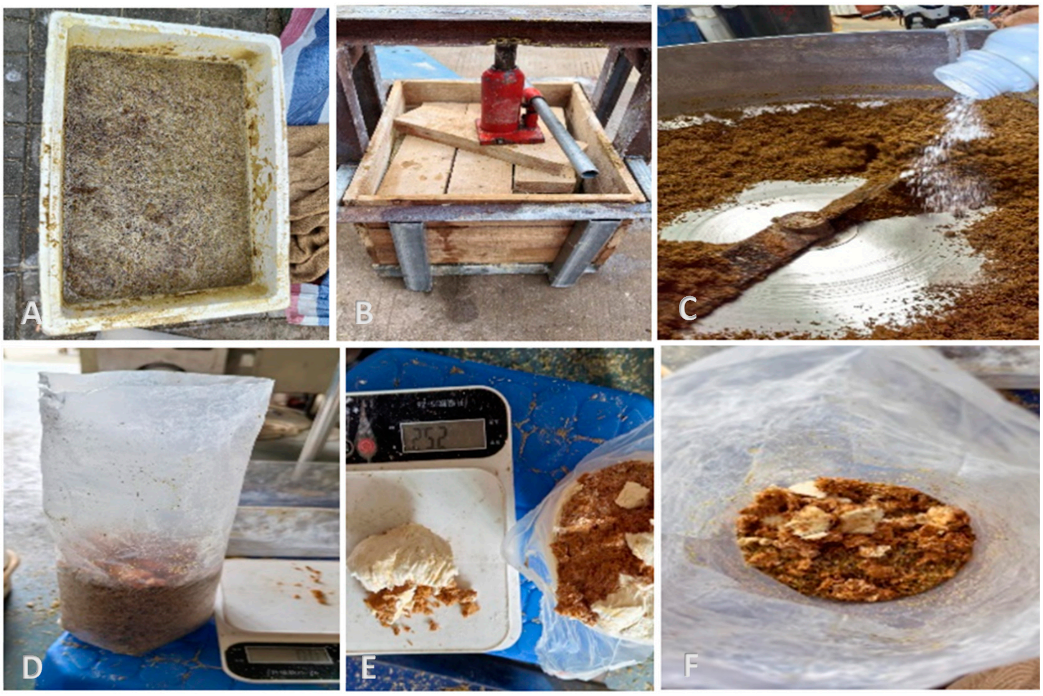

Figure S5. Stepwise procedure for NSOI preparation and inoculation. A; substrate soaked in 2% lime water, B: Squeezing out of excess water, C; Mixing and addition of 2% lime, D: Packing and weighing of 500g substrate, E; Weighing of 5% spawn, and F; Inoculation

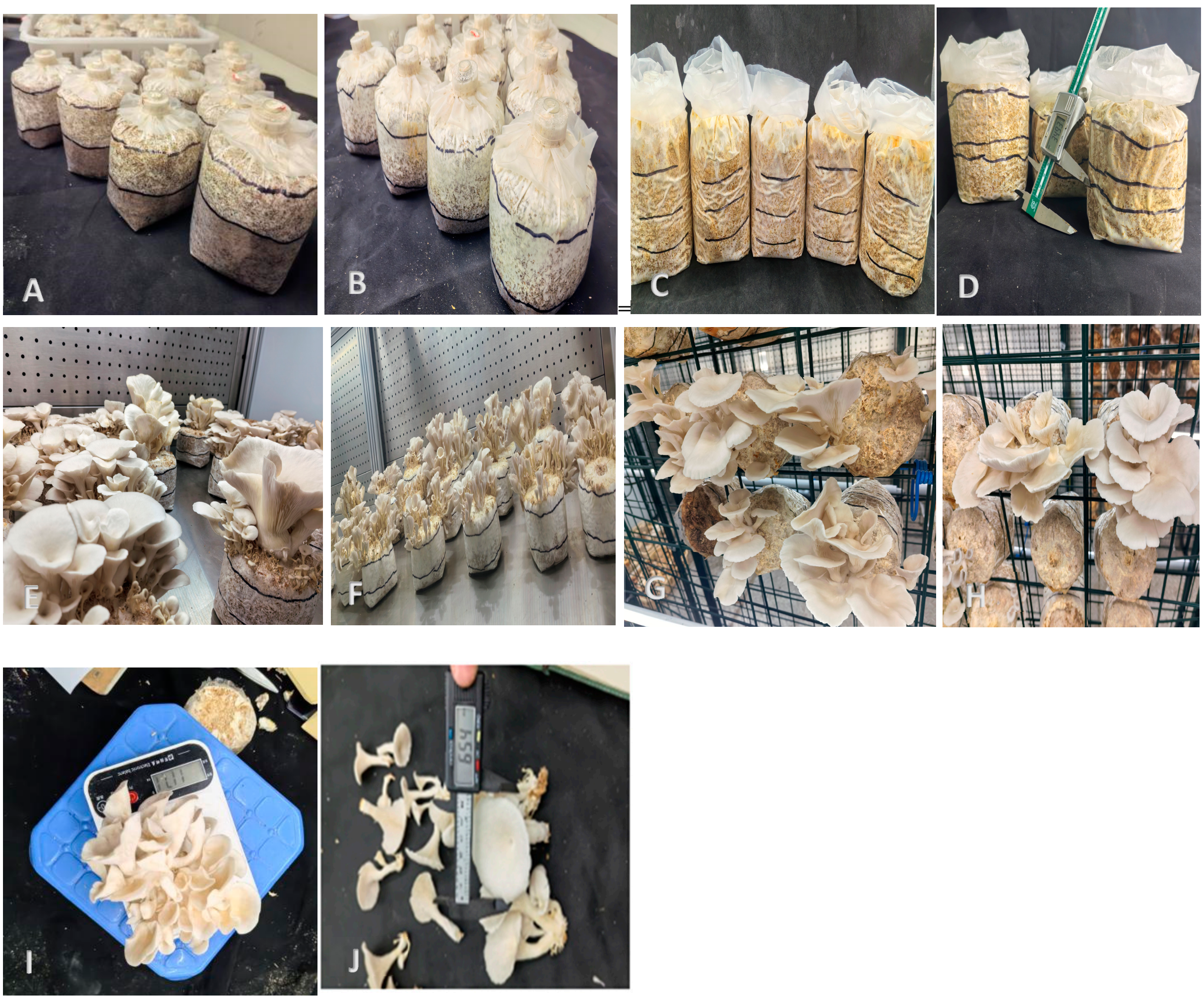

Figure S6. Mycelium growth to fruiting and harvesting stages.

A, 2 weeks mycelium run for T1; B, 2 weeks mycelium run for T2; C, 4 weeks mycelium run for T3; D, 4 weeks mycelium run for T4; E, fruiting stage for T1; F, fruiting stage for T2; G, fruiting stage for T3; H, fruiting stage for T4; I, weighing of fruiting bodies (similar for all harvested treatments); J, measuring of physical structure (similar for all harvested treatments).

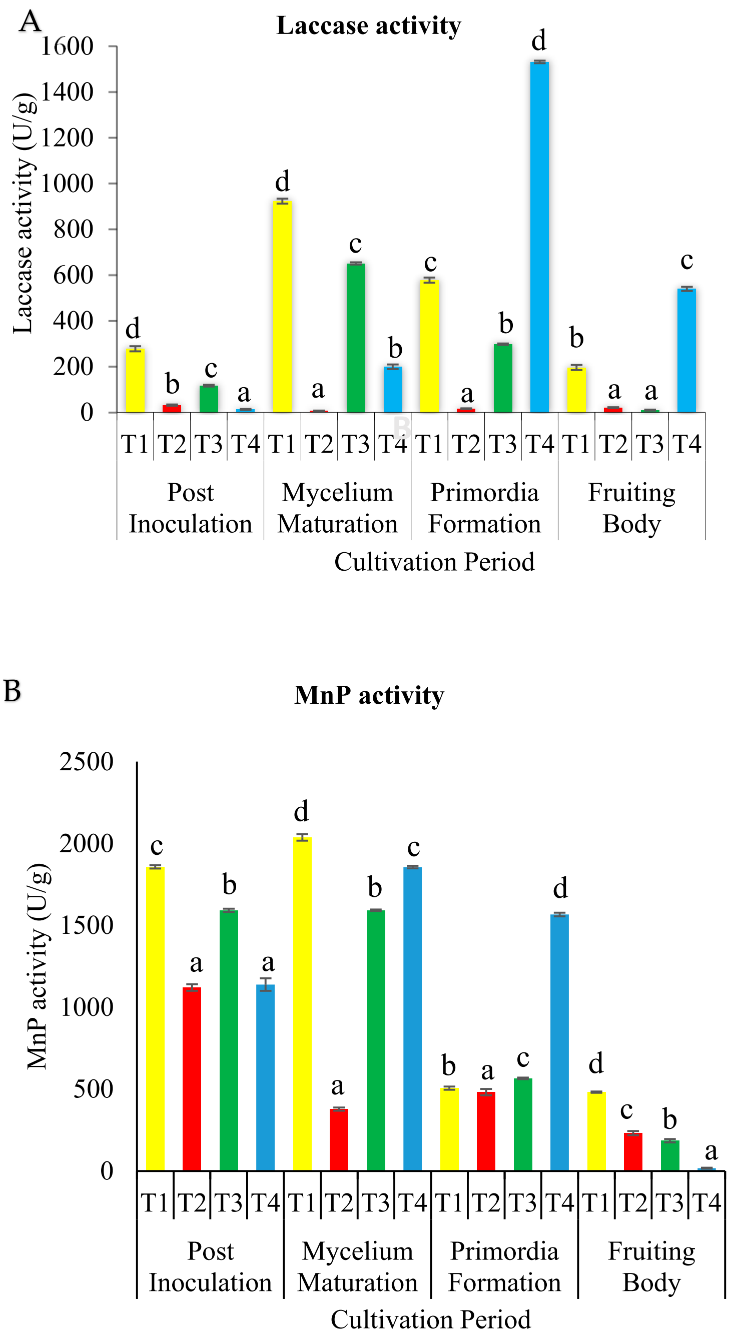
A
Laccase activity
Laccase activity (U/g)
0
200
400
600
800
1000
1200
1400
1600
d b c a d a c b c a b d b a a c
T1 T2 T3 T4 T1 T2 T3 T4 T1 T2 T3 T4 T1 T2 T3 T4
Post Inoculation
Mycelium Maturation
Primordia Formation
Fruiting Body
Cultivation Period
B
MnP activity
MnP activity (U/g)
0
500
1000
1500
2000
2500
c a b a d a b c b a c d d c b a
T1 T2 T3 T4 T1 T2 T3 T4 T1 T2 T3 T4 T1 T2 T3 T4
Post Inoculation
Mycelium Maturation
Primordia Formation
Fruiting Body
Cultivation Period

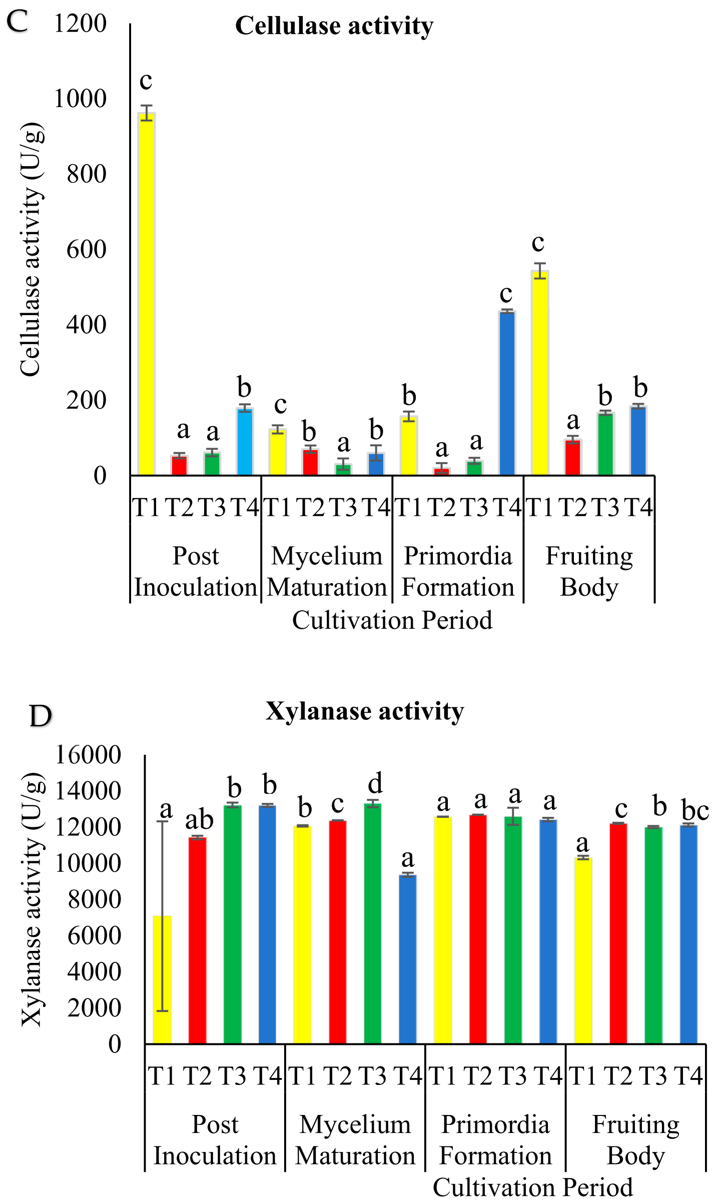
C
Cellulase activity
Cellulase activity (U/g)
1200
1000
800
600
400
200
0
c
a a b c b a b b a a c c a b b
T1 T2 T3 T4 T1 T2 T3 T4 T1 T2 T3 T4 T1 T2 T3 T4
Post Inoculation
Mycelium Maturation
Primordia Formation
Fruiting Body
Cultivation Period
D
Xylanase activity
Xylanase activity (U/g)
16000
14000
12000
10000
8000
6000
4000
2000
0
a ab b b b c d a a a a a a c b bc
T1 T2 T3 T4 T1 T2 T3 T4 T1 T2 T3 T4 T1 T2 T3 T4
Post Inoculation
Mycelium Maturation
Primordia Formation
Fruiting Body
Cultivation Period

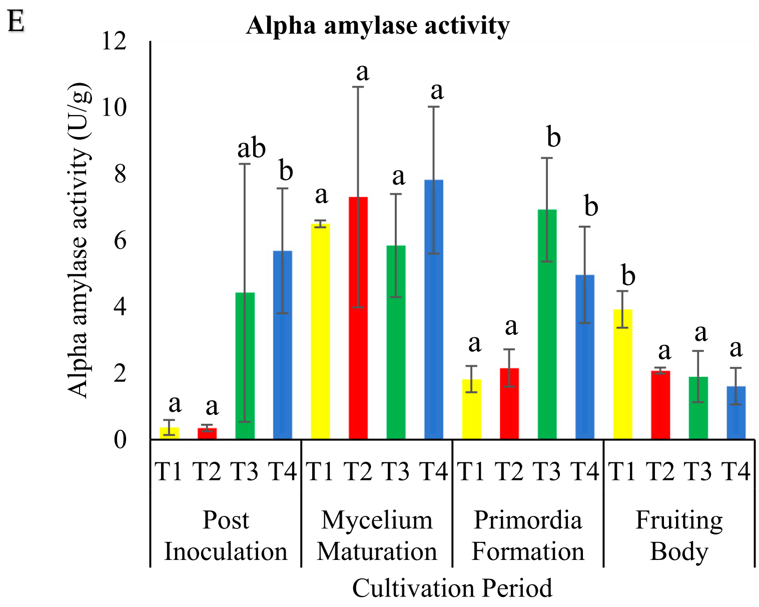

Figure S7. Comprehensive visualization of extracellular enzyme activities and metabolomic profiles of *P. ostreatus* PXF9 across different disinfection treatments. (A) Laccase activity; (B) Manganese peroxidase (MnP) activity; (C) Cellulase activity; (D) Xylanase activity; (E) Alpha-amylase activity. Enzyme activities were assessed at four growth stages: post-inoculation (PI), mycelium maturation (MM), primordia formation (PF), and fruiting body (FB). Different letters above bars indicate significant differences at $p < 0.05$ according to Duncan's multiple range test. Treatment codes: T1, CSAI (experimental); T2, CSAI (control); T3, SSOI; T4, NSOI.

Supplementary Tables

Table S1. Composition of Potato Dextrose Agar (PDA).

**Composition**	**weight (g)**
Potato infusion	200
Dextrose	20
Agar	20
Distilled water	1 000

Table S2: Total material costs per 30 bags

**Cultivation group**	**Cost (USD)**
CSAI (T1, T2)	2.05 (bags) + 0.08 (spawn) + 0.34 ($CaCO_3$) + 2.72 (wheat bran) = 5.19
SSOI (T3)	2.05 (bags) + 0.21 (spawn) + 0.68 ($CaCO_3$) + 2.72 (wheat bran) = 5.66
NSOI (T4)	2.05 (bags) + 0.21 (spawn) + 2.03 (quicklime) + 2.72 (wheat bran) = 7.01

Table S3. OPLS-DA Model Parameters and Validation.

Comparison	Component	N	R^2X(cum)	R^2Y(cum)	Q^2(cum)	Q^2	Validation Status

	s (A)					Intercept	
B vs A	1+1+0	6	0.703	1.0	0.989	-0.32	Validated
B vs C	1+1+0	6	0.676	1.0	0.991	-0.30	Validated
B vs D	1+1+0	6	0.712	1.0	0.987	-0.35	Validated
B vs A	1+1+0	6	0.703	1.0	0.989	-0.32	Validated

OPLS-DA model parameters for pairwise group comparisons. A represents the number of components (predictive + orthogonal + negative); N indicates the number of observations (samples). R^2X(cum) represents the cumulative explained variance in X (metabolite data); R^2Y(cum) represents the cumulative explained variance in Y (group membership); Q^2(cum) indicates predictive ability determined through 7-fold cross-validation. Negative Q^2 intercepts from 200-permutation testing confirm model validity and absence of overfitting in all comparisons.
